# Cooled radiofrequency ablation of genicular nerves provides 24‐Month durability in the management of osteoarthritic knee pain: Outcomes from a prospective, multicenter, randomized trial

**DOI:** 10.1111/papr.13139

**Published:** 2022-06-29

**Authors:** Jeffrey Lyman, Fred Khalouf, Keith Zora, Michael DePalma, Eric Loudermilk, Maged Guiguis, Douglas Beall, Lynn Kohan, Antonia F. Chen

**Affiliations:** ^1^ Institute for Orthopedic Research and Innovation Coeur d'Alene Idaho USA; ^2^ University Orthopedics Center Altoona Pennsylvania USA; ^3^ University Orthopedics Center State College Pennsylvania USA; ^4^ Virginia iSpine Physicians Richmond Virginia USA; ^5^ University of Virginia School of Medicine Charlottesville Virginia USA; ^6^ Ochsner Clinic Foundation New Orleans Louisiana USA; ^7^ Clinical Investigations Edmond Oklahoma USA; ^8^ PCPMG Clinical Research Unit LLC Anderson South Carolina USA; ^9^ Department of Orthopaedics, Brigham and Women's Hospital Boston Massachusetts USA

**Keywords:** chronic knee pain, cooled radiofrequency ablation, genicular neurotomy, osteoarthritis

## Abstract

**Objective:**

To assess long‐term outcomes of cooled radiofrequency ablation (CRFA) of genicular nerves for chronic knee pain due to osteoarthritis (OA).

**Methods:**

A prospective, observational extension of a randomized, controlled trial was conducted on adults randomized to CRFA. Subjects were part of a 12‐month clinical trial comparing CRFA of genicular nerves to a single hyaluronic injection for treatment of chronic OA knee pain, who then agreed to visits at 18‐ and 24‐months post CRFA and had not undergone another knee procedure since. The subjects were evaluated for pain using the Numeric Rating Scale (NRS) function using the Western Ontario and McMaster Universities Osteoarthritis Index (WOMAC), subjective benefit using the Global Perceived Effect (GPE) scale, quality of life using the EuroQol‐5‐Dimensions‐5 Level (EQ‐5D‐5L) questionnaire, and safety.

**Results:**

Of 57 subjects eligible, 36 enrolled; 32 completed the 18‐month visit with a mean NRS score of 2.4 and 22 (69%) reporting ≥50% reduction in pain from baseline (primary endpoint); 27 completed the 24‐month visit, with a mean NRS of 3.4 and 17 (63%) reporting ≥50% pain relief. Functional and quality of life improvements persisted similarly, with mean changes from baseline of 53.5% and 34.9% in WOMAC total scores, and 24.8% and 10.7% in EQ‐5D‐5L Index scores, at 18‐ and 24‐months, respectively. There were no identified safety concerns in this patient cohort.

**Conclusion:**

In this subset of subjects, CRFA of genicular nerves provided durable pain relief, improved function, and improved quality of life extending to 24 months post procedure, with no significant safety concerns.

**Key Points**

- Cooled radiofrequency ablation provides extended clinical benefit in the management of knee osteoarthritis.
- Subjects experienced meaningful improvements in both pain and function lasting 24 months following a single treatment.

## INTRODUCTION

Osteoarthritis (OA) is a painful and debilitating chronic degenerative disease of the joints, which commonly affects the knees and limits mobility.^1^ Although total knee arthroplasty is well‐established as a definitive treatment for late‐stage knee OA, associated risks include acute and chronic post‐operative pain, infection, blood clots, and even death.^2^ Moreover, patients with knee OA typically suffer from pain for many years before becoming surgical candidates.

Aside from systemic pharmacotherapies with their own side effect burdens, there are few alternative treatments for symptomatic management of osteoarthritic knee pain. Intra‐articular steroid injection (IAS) provides short‐term pain relief, but must be repeated often, risking increased joint cartilage destruction.^3^ Hyaluronic acid (HA) has some supporting evidence^4^ but has failed to earn the recommendation of the American Academy of Orthopedic Surgeons.^5^ Platelet‐rich plasma, prolotherapy, and stem cell therapy have gained increasing attention but still lack substantial evidence.^6^

Radiofrequency ablation (RFA) is the targeted thermal damage of sensory nerves to interrupt the transmission of pain signals, such that pain is attenuated while the nerves regenerate. RFA generates heat within tissues by passing alternating electric current between the unshielded electrode at the tip of a radiofrequency probe and a distant ground electrode on the patient's skin; the agitation of ionic species near the probe tip causes friction and heat, leading to localized tissue destruction. The radiofrequency (RF) probe tip is heated indirectly by conduction of heat from the target tissue. In standard RFA, tissues adjoining the probe tip can reach 80°C, at which point further RF energy transfer may be limited by a loss of conductivity that occurs if the tissue desiccates or chars, thereby becoming a high‐impedance insulator. Cooled RFA (CRFA) overcomes this limitation because water circulated within the probe tip keeps the probe tip‐tissue interface at 60°C, enabling electric current to penetrate more deeply to create larger, spherical lesions by effectively projecting the zone of tissue ablation beyond the probe tip,^7^ as temperatures beyond the tissue‐tip interface reach 80°C.^8^

CRFA of joint‐specific sensory nerves has been shown to provide at least 12 months of relief for painful conditions of the spine,^9^, ^10^, ^11^ and has gained recognition as a minimally invasive treatment option for pain associated with knee OA.^12^ A prospective, multicenter, randomized, crossover trial involving 151 subjects with chronic knee pain (duration ≥6 months) unresponsive to conservative modalities compared CRFA of genicular nerves to a single IAS injection: at 6 months post intervention, 74% of CFRA subjects reported ≥50% reduction in pain from baseline compared to just 16% in the IAS group (*p* < 0.0001);^13^ at 12 months post intervention, 65% of CRFA subjects maintained ≥50% pain relief, with a mean overall reduction of 4.3 Numeric Rating Scale (NRS) points from baseline (*p* < 0.0001) and substantial functional improvements.^14^

The sustained benefits of genicular CFRA observed by Davis et al.^14^ prompted the investigators to enroll CFRA subjects from the 12‐month controlled trial in an observational extension study, the results of which showed sustained pain relief and functional improvements persisting to 18 and 24 months after CRFA.^15^ Meanwhile, study investigators conducted a second prospective, multicenter, randomized, crossover trial—this time comparing CRFA of genicular nerves to viscosupplementation with a single intra‐articular injection of HA in 177 adults with chronic knee pain due to OA.^16^ At 6 months post intervention, 71% of the CRFA subjects reported ≥50% reduction in pain from baseline compared with just 38% in the HA group (*p* < 0.0001). At 12 months post procedure, 65% of the original CRFA subjects met the primary endpoint of ≥50% pain relief from baseline, and 64% reported improved knee condition by Global Perceived Effect (GPE); CRFA subjects collectively realized a 46.2% improvement in total Western Ontario and McMaster Universities (WOMAC) score, and all reported improved quality of life due to the procedure. Safety profiles for CRFA and HA were similar and in line with expectations for each product.^17^ Thus, the purpose of this study was to expand the patient cohort for long‐term outcomes of CRFA of genicular nerves for chronic knee pain due to OA.

## METHODS

### Subjects

The current study was a planned extension of the Chen et al. (2020) controlled trial, designed to follow a sample of subjects to 18‐and 24‐months post CRFA timepoints to assess the durability of the 12‐month efficacy and safety of genicular CRFA for treatment of chronic OA knee pain.^16^, ^17^

In the original controlled trial, a baseline NRS score of ≥6 (usual daily pain) for the index knee was required for enrollment in the controlled trial, as was a baseline of score of 2 or 3 on WOMAC question A1 (pain while walking on flat surface) and a baseline mean score of 1.5 to 3.5 on all five questions of the WOMAC subscale A (pain). Subjects were randomized 1:1 to receive either CRFA (COOLIEF*, Avanos Medical, Inc., Alpharetta, GA, USA) or a single intra‐articular HA injection (Synvisc‐One® [hylan G‐F 20]; Sanofi) with follow‐up visits conducted at 1, 3, 6, and 12 months post intervention.

Prior to randomization, subjects underwent fluoroscopically guided blockade of four targets genicular nerves. Positive responders (defined as a ≥ 50% reduction in pain score in NRS) proceeded to randomization. Those undergoing CRFA received genicular ablation following previously published methods.^16^ Needles were advanced to the bony endpoint of each genicular target (superolateral portion of femoral condyle, superomedial portion of femoral condyle, inferomedial portion of tibial condyle, and midline of the femur, approximately 2 cm cephalad of the upper patellar border). This study differed from previously published studies,^13^ in that a fourth nerve target for ablation was added (the upper patellar).

Motor stimulation was conducted, followed by the injection of local anesthetic. CRFA of each of the target nerves was conducted with a probe set temperature of 60°C for 2 minutes and 30 seconds, which produced an average tissue temperature of greater than 80°C.

**FIGURE 1:**
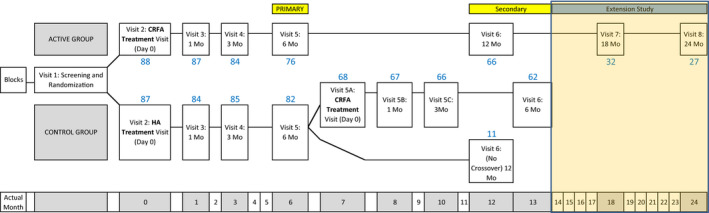
Context of 18‐ and 24‐month outcomes analysis within clinical trial design

After the 6‐month post intervention follow‐up visit, subjects dissatisfied with the HA treatment could cross over (XO) to CRFA. However, unlike Hunter et al. ^15^ only subjects originally treated with CRFA following randomization were eligible for the current extension to 18‐ and 24‐months post CRFA (Figure 1). Subjects were not required to attend both the 18‐ and the 24‐month visits that were to be included in the analysis.

While Chen et al.^16^, ^17^ treated 88 subjects randomized to CRFA across 10 study sites, the current observational extension study was conducted at just 7 of those 10 study sites. The study protocol, informed‐consent forms, subject recruitment materials, and study protocol amendments were approved by each center's institutional review board. Subjects provided informed consent for the controlled trial prior to the initiation of screening activities, and then again to enroll in the extension. This trial was registered in ClinicalTrials.gov (NCT03381248) prior to initiation.

### Study outcomes

Patient assessments were made primarily during in‐office visits or by telephone in the event of COVID‐19 restrictions or other confounding issues as described in Figure 3. For in‐office visits, subjects completed questionnaires without assistance from study staff. For telephone visits, study coordinators posed the questions or prompts and recorded each subject's verbal responses.

Consistent with the original controlled trial, the primary efficacy endpoint for the observational extension study was the proportion of subjects (“responders”) whose usual daily knee pain was reduced by ≥50% from baseline at 18 and 24 months after CRFA. At each visit, subjects rated their usual knee pain using an 11‐point NRS, with zero indicating no pain and 10 indicating the worst pain ever,^18^ in the following categories: least pain, worst pain, pain right now, and usual daily pain—all according to subject's impression for the week preceding data collection. Raw scores were averaged to calculate the category group mean at each time point.

The WOMAC^19^ Osteoarthritis Index was used to evaluate subjects' overall knee function over the previous 48 hours; change in the WOMAC total score from baseline was a secondary endpoint in the controlled trial and extension. The WOMAC is a self‐administered 24‐item questionnaire divided into three subscales: pain (5 items), stiffness (2 items), and physical function (17 items). Each item is scored 0–4: None (0) to Extreme (4). Higher scores indicate worse pain, stiffness, and functional limitations. Subjects either completed the questionnaire at in‐person visits or answered the items posed by research staff over the telephone.

Subjects rated the impact of genicular CFRA on their quality of life using tertiary outcome measures including the Global Perceived Effect (GPE) scale^20^ and the EuroQol‐5 Dimensions‐5 Level (EQ‐5D‐5L) health‐related quality of life questionnaire^21^ at baseline (except GPE) and at 1, 3, 6, and 12 months following CRFA in the controlled trial, and at the 18‐ and 24‐month post CRFA visits in the extension. The GPE scale is a quality of life instrument used to assess a subject's global assessment of change in their chief complaint after receiving treatment on a seven‐point scale, where 1 = worst ever and 7 = best‐ever.

The EQ‐5D‐5L questionnaire consists of two pages: (a) a descriptive system section that asks patients to rate the current intensity level of their problems (none, slight, moderate, severe, and extreme) in five dimensions of health including mobility, self‐care, usual activities, pain/discomfort, and anxiety/depression; 1‐digit numbers that code the intensity level for each of the five dimensions can then be combined into a 5‐digit number that describes the patient's health state and (b) a second section (EQ Visual Analogue Scale [VAS]) that asks subjects to rank their overall health status on a VAS from 0 to 100, where 0 = worse than death and 100 = state of perfect health. Both the GPE and the EQ‐5D‐5L related to the respondent's immediate situation at the time of completion (ie “today”).

At each study visit, subjects were evaluated for concomitant medications, as well as adverse events and serious adverse events—the primary safety endpoint. In addition, demographic data and medical history were reviewed and amended. Radiographic analysis was conducted in extension study subjects at the 24‐month post CRFA visit to monitor for changes in grade of OA as measured by the Kellgren‐Lawrence scale.^22^

### Statistical analysis

Data management, study site monitoring, and statistical analysis services were performed by a third party independent of Avanos Medical. Data were reported using descriptive statistics, including means, standard deviations (SDs), and 95% confidence intervals (CIs) for continuous outcomes; and counts, percentages, and 95% CIs for categorical outcomes. Data from the original controlled trial^16^, ^17^ were incorporated into this analysis for subjects enrolled in the extension (eg demographic information, treatment information, outcomes from previous visits, etc.) to consider a subject's full post CRFA patient experience.

A Kaplan‐Meier survivor analysis was performed using an individual subject's loss of pain relief, as measured by the reduction from baseline in the NRS falling below 50%, as the terminal event. The *p*‐value to indicate significance for the superiority for NRS‐measured primary outcome of knee pain (≥ 50% relief compared to baseline pain) was 0.025, while that of all other presented outcomes was 0.05 [16 appendix]. In addition, logistic regression analysis was performed to identify possible predictors of pain—including baseline OA grade, duration of diagnosis, age, and baseline opioid use.

## RESULTS

### Subject disposition

The original controlled trial treated 88 subjects randomized to CRFA (at visit #2) across 10 study sites.^16^ Of these, 66 completed the 12‐month follow‐up visit.^17^ Of the 66 completers, 9 were located at study sites not participating in the current extension study, leaving 57 subjects eligible for the extension. Of the 57 eligible, 21 (37%) declined participation and 36 (63%) were enrolled and signed informed consent.

**FIGURE 2:**
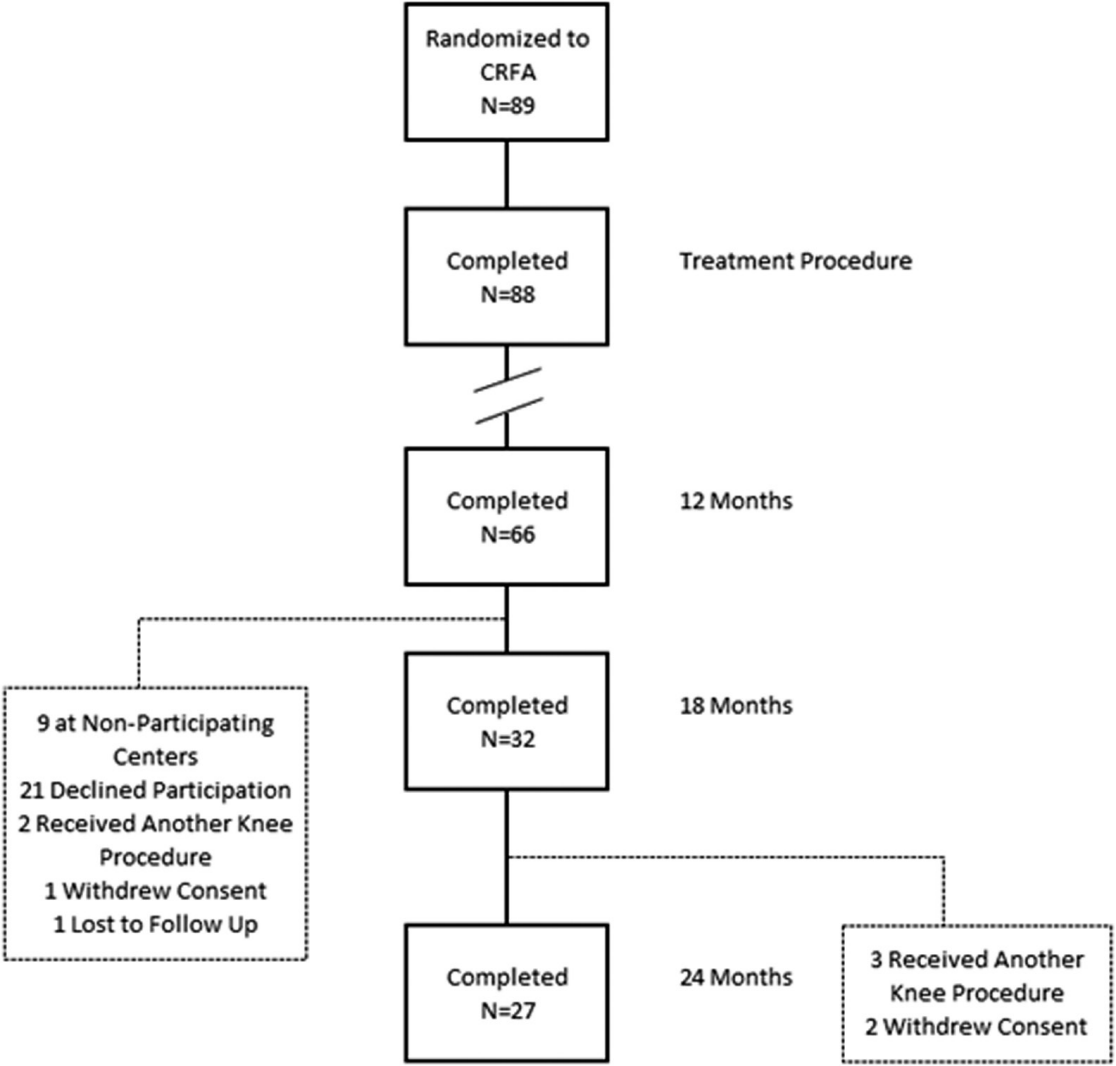
Consolidated Standards for Reporting (CONSORT) diagram of subject disposition. CRFA = cooled radiofrequency ablation

Of the 36 subjects enrolled in the extension study, 32 (89%) completed the 18‐month follow‐up visit, two had disqualifying knee procedures, one withdrew consent, and another was lost to follow‐up (Figure 2). Twenty‐seven remaining subjects were evaluated again at 24 months after CRFA treatment, while 2 withdrew consent, and 3 received a disqualifying index knee procedure: in each case IAS injection (*n* = 3) was administered at a mean (range) of 654 (622–688) days after CRFA.

Due to the 24‐month follow‐up visits falling after the onset of the COVID‐19 pandemic, 6 of the 27 subjects evaluated at 24 months post CRFA were evaluated remotely and 6 were evaluated outside the scheduled ±2‐week visit window (protocol deviation)—1 to 2 days before and the other five on average 18 days (max = 40 days) after the scheduled visit window (Table 1).

**TABLE 1:** COVID impact by visit type—extension study subjects

	18 Month	24 Month
Visit type [n/N (%)]		
In office	31/32 (96.9)	21/27 (77.8)
Remote	1/32 (3.1)	6/27 (22.2)
COVID impact [n/N (%)]^a^		
In office, out of window	0/32 (0.0)	4/27 (14.8)
Remote, missing assessment(s)	0/32 (0.0)	6/27 (22.2)
Remote, out of window	0/32 (0.0)	2/27 (7.4)
Days out of window		
N	–	6
Mean (SD)	–	17.8 (17.1)
Median	–	18.5
Min. Max.	–	−2.0, 40.0

^a^ (not mutually exclusive).

### Primary outcome: knee pain

**FIGURE 3:**
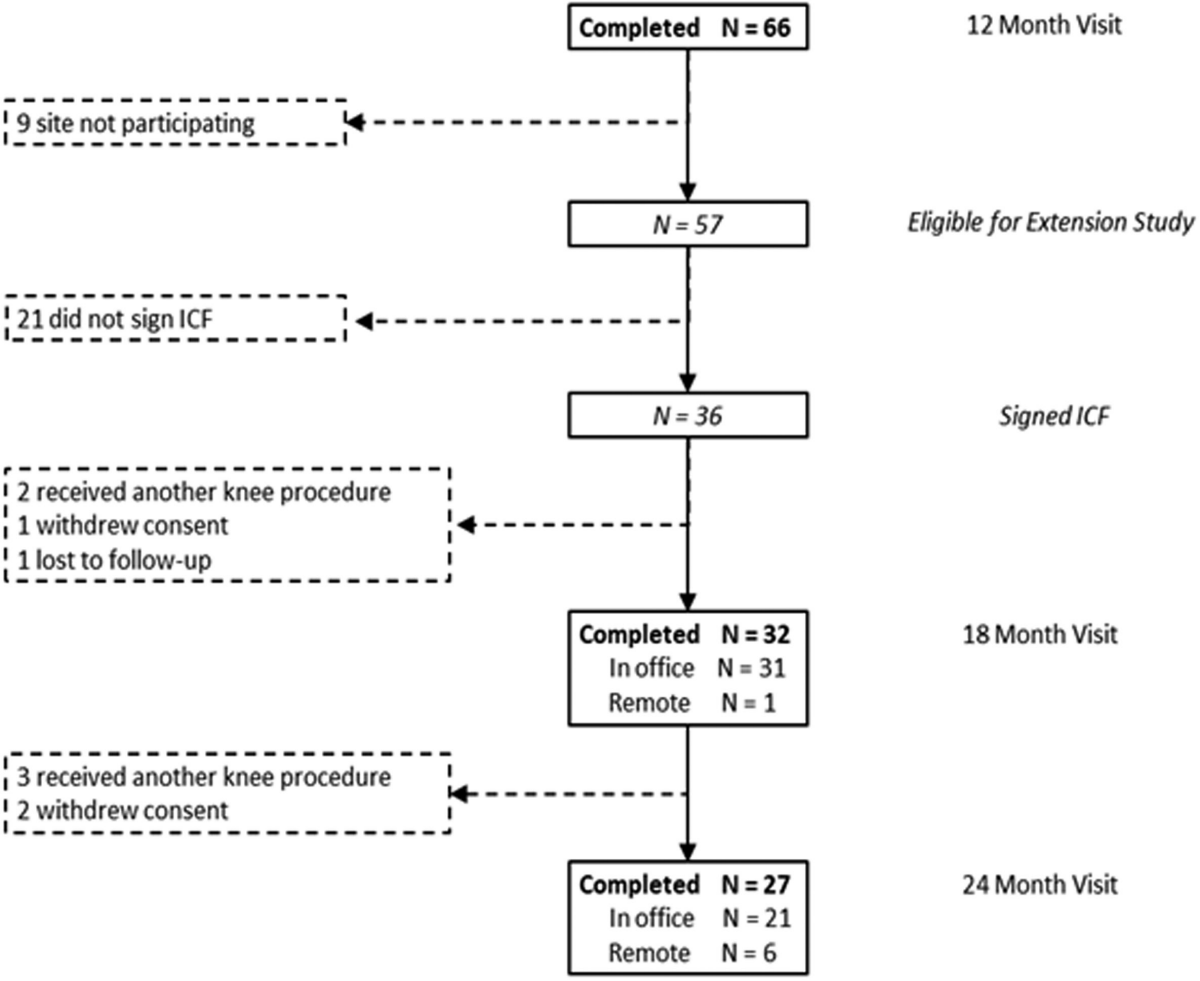
Subject disposition—extension phase. ICF, Informed Consent Form

Table 2 and Figure 3 contain longitudinal data specifically from the subjects in the extension study. Mean (±SD) NRS pain scores for CRFA extension subjects were significantly decreased (*p* < 0.0001), from 6.8 ± 0.8 at baseline to 2.4 ± 2.5 and 3.4 ± 3.2 at 18‐ and 24‐months post CRFA visits, respectively. Results indicated that 22/32 subjects (69%) at 18 months and 17/27 subjects (63%) at 24 months post CRFA continued to experience at least 50% reduction in pain from baseline. Results from logistic regression analysis on possible predictors of pain—including baseline OA grade, duration of diagnosis, age, and baseline opioid use—did not identify any significant predictors of pain in terms of NRS ratings at either 18‐ or 24‐months post CRFA visits.

**TABLE 2:** Numeric rating scale results

	Baseline	1 Months	3 Months	6 Months	12 Months	18 Months	24 Months
	*n =* 32^b^	*n =* 32^b^	*n =* 32^b^	*n =* 32^b^	*n =* 32^b^	*n =* 32	*n =* 27
Numeric rating scale^a^ ^,^ ^b^							
Mean ± SD	6.8 ± 0.8	2.8 ± 2.6	2.0 ± 2.1	2.1 ± 2.0	1.9 ± 1.9	2.4 ± 2.5	3.4 ± 3.2
95% CI for the mean	6.5 to 7.1	1.9 to 3.8	1.3 to 2.7	1.4 to 2.8	1.2 to 2.6	1.5 to 3.4	2.1 to 4.7
Change from baseline (%)^a^ ^,^ ^b^							
Mean ± SD	–	58.7 ± 39.1	71.1 ± 29.1	69.3 ± 29.5	71.9 ± 27.5	64.1 ± 36.9	50.7 ± 46.0
95% CI for mean	–	4.6 to 72.8	60.6 to 81.5	58.7 to 80.0	62.0 to 81.8	50.8 to 77.4	32.5 to 68.9
≥50% Improvement *n* (%)		22 (68.8)	26 (81.3)	24 (75.0)	23 (71.9)	22 (68.8)	17 (63.0)

^a^ Data are presented as mean and standard deviation (SD) along with 95% confidence interval (CI).

^b^ Data from the controlled trial were included from baseline to 12 months for subjects enrolled in this extension study.

### General knee condition following CRFA

For subjects enrolled in the extension study, the general condition of the index knee—based on WOMAC total scores—improved from a baseline mean (±SD) of 64.4 ± 14.7 to 27.7 ± 21.8 at 3 months post CRFA and then remained stable through 18 months post CRFA, landing at 29.3 ± 25.3 (*p* < 0.0001; Table 3), for a mean improvement of 54%. Then at 24 months post CRFA, the mean WOMAC total score increased to 41.3 ± 29.9, but continued to reflect significantly improved knee pain, stiffness, and function (35%) relative to baseline (*p* = 0.0007).

**TABLE 3:** WOMAC knee score results

	Baseline	1 Months	3 Months	6 Months	12 Months	18 Months	24 Months
	*n =* 32^b^	*n =* 32^b^	*n =* 32^b^	*n =* 32^b^	*n =* 32^b^	*n =* 32	*n =* 27
WOMAC total score^a^ ^,^ ^b^							
Mean ± SD	64.4 ± 14.7	34.1 ± 23.8	27.7 ± 21.8	28.4 ± 21.2	27.4 ± 23.2	29.3 ± 25.3	41.3 ± 29.9
95% CI for the mean	59.1to 69.7	25.6 to 42.7	19.8 to 35.5	20.8 to 36.1	19.1 to 35.8	19.9 to 38.8	29.2 to 53.4
Change from baseline^a^ ^,^ ^b^							
Mean ± SD	–	30.3 ± 23.9	36.7 ± 25.1	36.0 ± 22.2	36.9 ± 25.2	34.7 ± 27.5	24.8 ± 32.8
95% CI for mean	–	21.6 to 38.9	27.7 to 45.8	28.0 to 44.0	27.9 to 46.0	24.4 to 44.9	11.6 to 38.1
P‐value		<0.0001^c^	<0.0001^c^	<0.0001^c^	<0.0001^c^	<0.0001^c^	0.0007^c^
WOMAC % Improvement from baseline		47.4	55.5	55.9	56.5	53.5	34.9

^a^ Data are presented as mean and standard deviation (SD) along with 95% confidence interval (CI).

^b^ Data from the controlled trial were included from baseline to 12 months for subjects enrolled in the extension study.

^c^ Paired t‐test.

### Global perceived effect

**FIGURE 4:**
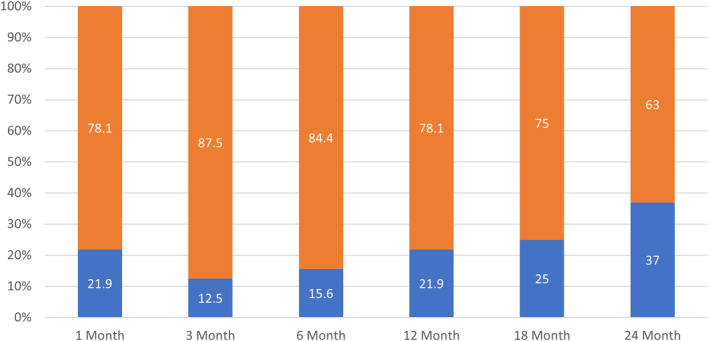
Binary distribution of Global Perceived Effect (GPE) Score†. 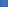 Not improved/worse 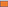 Improved. † Data from the original controlled trial^16^, ^17^ included from baseline to 12 months post cooled radiofrequency ablation for subjects enrolled in this study

Data collected using the GPE scale were later transformed during analysis to a binary measure—“improved” (5 to 7) or “not improved/worse” (1–4)—to ease interpretation. The resulting data revealed that 75% of extension subjects (24/32) reporting data at 18 months post CRFA, and 63% of subjects (17/27) at 24 months post CRFA, indicated a persistent improvement in their knee pain condition (Figure 4).

### EQ‐5D‐5L

Extension subjects reported sustained improvement in general health and quality of life following CRFA based on EQ‐5D‐5L Index scores, with mean increases from baseline of 0.15 points (*p* < 0.0001) at 18 months and 0.07 points (*p* = 0.0146) at 24 months post CRFA (Table 4). The difference at 18 months more than doubled the minimal clinically important difference (MCID) of 0.074 points on EQ‐5D‐5L Index, while the residual improvement at 24 months nearly matched the MCID for that measure.^21^

**TABLE 4:** Global perceived effect

	1 Months	3 Months	6 Months	12 Months	18 Months	24 Months
	*n =* 32^b^	*n =* 32^b^	*n =* 32^b^	*n =* 32^b^	*n =* 32	*n =* 27
Distribution of global perceived effect score^a,b^						
Not improved/worse	7 (21.9)	4 (12.5)	5 (15.6)	7 (21.9)	8 (25.0)	10 (37.0)
Improved	25 (78.1)	28 (87.5)	27 (84.4)	25 (78.1)	24 (75.0)	17 (63.0)

^a^ Data are presented as number of subjects (%) or 95% exact binomial confidence interval (CI).

^b^ Data from the original study were included from baseline to 12 months for subjects enrolled in this extension study.

### Radiographic evaluation

Radiographic examination was required as part of follow‐up in the extension study only at 24 months post CRFA timepoint. Mainly due to COVID‐19 pandemic‐related reduction in office assessments, only 22 of 27 subjects had radiographic exams at 24 months post CRFA. Compared with their own individual baseline radiographic evaluations from 2 years earlier, 68.2% (15/22) demonstrated no change in Kellgren‐Lawrence grade, 22.7% (5/22) showed worsening of 1 OA grade, and 9.1% of subjects (2/22) showed worsening by 2 grades.^22^ These results were highly consistent with those reported by Chen et al.^17^ for many of the same subjects at 12 months post CFRA 1 year earlier, at which time 87.5% (28/32) of subjects showed no change and 12.5% (4/32) demonstrated worsening of 1 OA grade (Table 5).

**TABLE 5:** Radiographic evaluation—extension study subjects

	Baseline^a^	12 Month^a^	24 Month^a^
	*n* = 32^b^	*n* = 32^b^	*n* = 22
Radiographic evaluation^c^			
Grade 1 OA/ None	0 (0.0)	0 (0.0)	0 (0.0)
Grade 2 OA/Mild	5 (15.6)	3 (9.4)	1 (4.5)
Grade 3 OA/Moderate	15 (46.9)	15 (46.9)	7 (31.8)
Grade 4 OA/Severe	12 (37.5)	14 (43.8)	14 (63.6)
Change from baseline in radiographic evaluation			
Worsening of 2 grades	–	0 (0.0)	2 (9.1)
Worsening of 1 grade	–	4 (12.5)	5 (22.7)
No change	–	28 (87.5)	15 (68.2)
Improvement of 1 grade	–	0 (0.0)	0 (0.0)
Improvement of 2 grades	–	0 (0.0)	0 (0.0)

^a^ Data are presented as number of subjects (%).

^b^ Data from the original study were included at baseline and 12 months for subjects enrolled in this study.

^c^ Kellgren‐Lawrence grade.

### Kaplan‐meier survivor analysis

**FIGURE 5:**
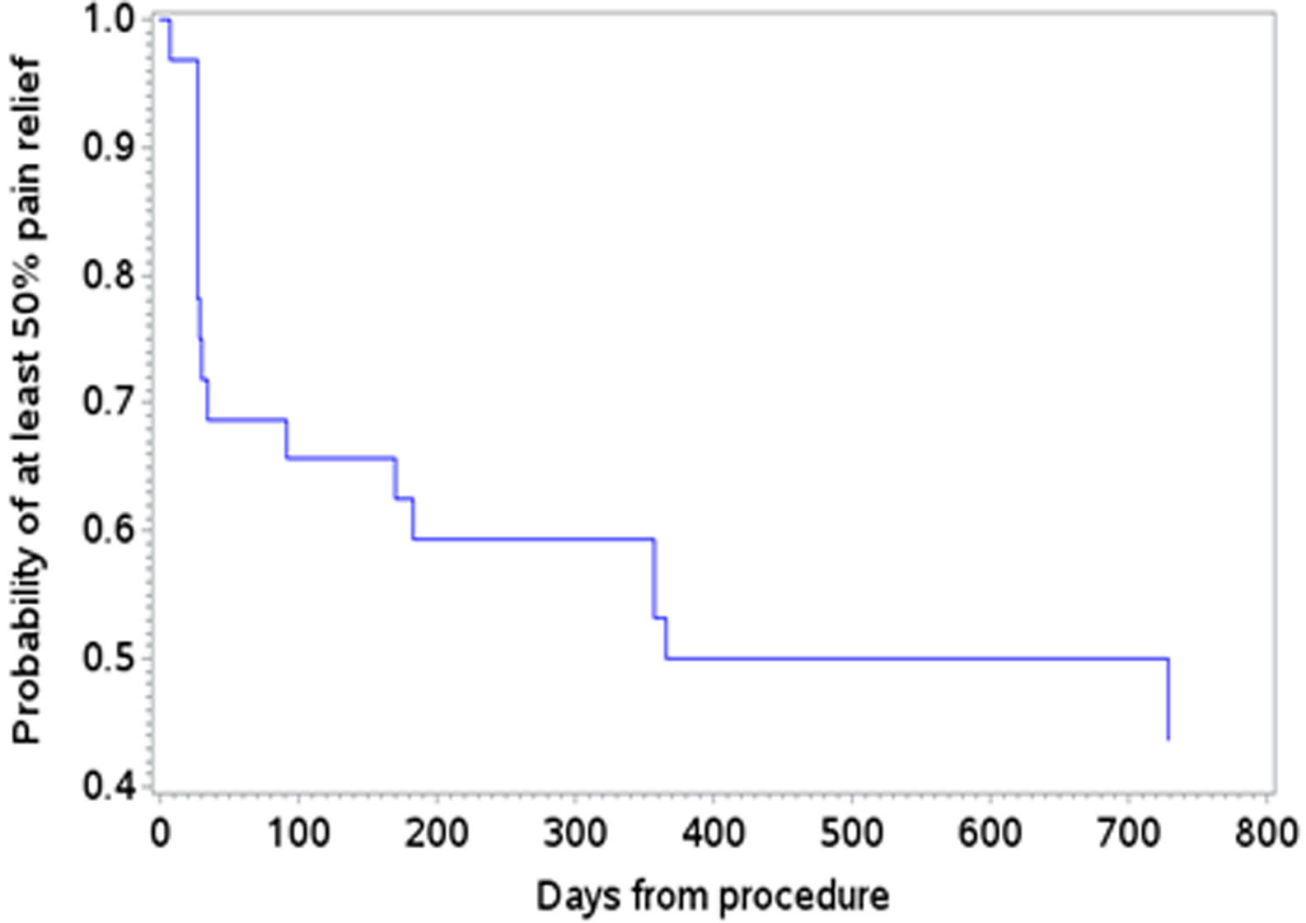
Survival to first occurrence of <50% pain relief—extension study subjects†. †Data from the original controlled trial included from baseline to 12 months for subjects enrolled in this study

Figure 5 depicts the Kaplan‐Meier survivor curve plotted using the end of an individual patient's pain relief—as measured by reduction in the NRS from baseline falling below 50%—as the terminal event. This analysis suggests that the small subset of patients in this study had an approximately 50% chance of maintaining 50% or greater relief of OA knee pain through 700 days after genicular CRFA.

### Adverse events

There were no serious or nonserious adverse events related to the CRFA procedure reported at 18 and 24 months following CRFA. Adverse events reported up to 12 months post CRFA were detailed in previous publications.^16^, ^17^

## DISCUSSION

The confluence of the global coronavirus pandemic's impact on elective surgery and reaction to the ongoing opioid crisis has focused demand for prospective research on durable non‐surgical methods for managing knee OA pain and disability.^23^, ^24^ While the safety and effectiveness of CRFA of genicular nerves in relieving pain and improving joint function in patients with knee OA is well established,^13^, ^25^, ^26^ data on outcomes beyond 12 months have been limited to one observational study by Hunter et al.^15^ involving subjects from the randomized crossover trial by Davis et al.^14^ comparing genicular CRFA with a single IAS injection for chronic OA knee pain.

The current study was informed by Hunter et al.^15^ but designed from the start as an observational extension of another randomized, multicenter, controlled crossover clinical trial comparing genicular CRFA with HA.^16^, ^17^ Whereas Hunter et al.^15^ included some crossover subjects initially treated with IAS injection followed by CRFA 6 months later for residual symptoms, the current study limited eligibility to subjects originally randomized to genicular CRFA. While the Davis^13^, ^14^ and Chen^16^, ^17^ randomized controlled trials were structurally similar, including one‐way elective crossover at 6 months, they differed in important aspects, including Chen's adding a fourth genicular target nerve, choice of active control, and secondary endpoints (eg WOMAC vs Oxford Knee Score). Despite these differences, the consistency and efficacy of treatment was similar across subjects within both trials.

One divergence deserving mention concerns the radiographic changes from baseline. Hunter^15^ did not require radiographic data of extension study for subjects completing assessments by telephone, so data were limited, but included multiple subjects whose Kellgren‐Lawrence grade^22^ improved from baseline, which Hunter credited against any CFRA‐related joint degeneration over the previous 18–24 months. By contrast, in the current study (Table 5), there was no apparent improvement in joint pathology observed at 24 months post CRFA. In fact, 68.2% (15/22) of subjects demonstrated no change in Kellgren‐Lawrence grade over 2 years, while 22.7% (5/22) saw worsening by 1 OA grade and 9.1% (2/22) saw worsening by 2 OA grades, which likely represented the natural progression of the disease.^27^

The durability of genicular CRFA in terms of pain relief was consistently mirrored by improvements in joint function and overall quality of life. For example, even at 24 months post CRFA, the mean 40% improvement in WOMAC pain score over baseline was at least double the 12% to 18% improvement in the WOMAC pain score from baseline, that is, MCID in patients with OA.^28^ Extension subjects reported sustained improvement in general health and quality of life following CRFA based on EQ‐5D‐5L Index scores, with a mean increase of 0.15 points at 18‐months and 0.07 points at 24 months post CRFA compared with baseline. The difference at 18 months post CRFA more than doubled the 0.074 point MCID on the EQ‐5D‐5L Index (Table 4) while the residual improvement at 24 months nearly matched the MCID.^21^

Even though HA‐treated positive control subjects were not followed beyond 12 months post CRFA,^17^ the 24‐month durability of genicular CRFA confirmed by this observational extension can be appreciated in terms of the HA comparison. Ong et al. recently devised an economic model to estimate the potential nationwide cost savings of using repeated doses of HA on OA patients to delay arthroplasty within the first 2 years of their illness. Based on a cohort of more than two million knee OA patients, investigators calculated a cost savings of $20,740 per patient based on 2017 costs.^29^ Given the superior efficacy and durability observed for CRFA in this study, cost savings of using CRFA instead of HA in this model may reasonably be estimated to be even greater. Additionally, previous retrospective trials have confirmed that CRFA maintains its effect in repeat treatment.^30^

Limitations of this study included the small sample size, with only a subset of patients enrolled in the trial being included in this analysis. It is not possible to draw broad conclusions from such a small sample size, but this study does provide insight to the clinical durability of CRFA in these subjects. There were a few minor protocol deviations due in part to the COVID‐19 pandemic, with subjects reporting data outside the predetermined follow‐up windows. However, collection of the primary and secondary data was not otherwise hindered, and there was no other reason to suspect the data collected were biased or incorrect. The lack of blinding due to the pragmatic study design admitted opportunities for bias.

Despite these limitations, findings from this study add important data to the literature reinforce previous findings that CRFA has the capability to provide sustained analgesia and functional improvement up to 24 months after a single application in patients suffering from OA knee pain.

## CONCLUSION

In this sample of patients randomized to genicular CRFA for chronic OA knee pain and followed prospectively, a majority experienced up to 24 months of pain relief, similar improvements in measures of function and quality of life, and no serious treatment‐related adverse events.

### ACKNOWLEDEMENTS

Medical writing assistance was provided by Avanos Medical.

## AUTHOR CONTRIBUTIONS

FK, KZ, MD, LK, MG, DB, EL, MP, IB, and JL participated in subject recruitment, enrollment and treatment. AC, LK, and JL contributed to the drafting and reviewing of the manuscript. All authors read and approved the final manuscript.

## CONFLICT OF INTERESTS

J.L. reports grants, personal fees, and non‐financial support from Avanos during the conduct of the study; F.K., K.Z., M.D., and E.L. have nothing to disclose. M.G. reports personal fees from Avanos Medical outside the submitted work. D.B. reports grants from Avanos. L.K. reports personal fees from Avanos Medical during the conduct of the study. A.C. reports personal fees from Avanos during the conduct of the study.

## Data Availability

The data that support the findings of this study are available on request from the corresponding author. The data are not publicly available due to privacy or ethical restrictions.
